# Developing a Community-Based Model for the Adoption of Sustainable Menstrual Products Among Adolescent Girls: Protocol for a 2-Phase Interventional Feasibility Study

**DOI:** 10.2196/93628

**Published:** 2026-09-22

**Authors:** Kapil Goel, Rekha Dutt, Madhu Gupta, Tanvi Kiran

**Affiliations:** 1 Department of Community Medicine and School of Public Health, Postgraduate Institute of Medical Education and Research, Chandigarh, India; 2 Department of Community Medicine and Family Medicine, All India Institute of Medical Sciences Kalyani, AIIMS, NH-34 Connector Road, Kalyani, West Bengal, 741245, India, 91 8961310911

**Keywords:** adolescent health, community health workers, health education, feasibility studies, menstrual hygiene products, menstrual cup

## Abstract

**Background:**

Adoption of sustainable menstrual products, such as menstrual cups (MCs) and menstrual panties (MPs), remains low in India despite national menstrual hygiene initiatives. Cultural taboos, low awareness, and misinformation are significant barriers. This study develops and evaluates a community-based intervention led by trained community health workers and female teachers to promote sustainable menstrual product use among adolescent girls.

**Objective:**

This study aimed to (1) assess the impact of sensitization and training on the knowledge, attitudes, and practices of community health and education workers; (2) evaluate the effectiveness of their community campaigns in improving girls’ awareness and readiness to adopt MCs and MPs; (3) examine the acceptability and user experience of MCs and MPs over 12 months; and (4) conduct an economic evaluation of MCs and MPs.

**Methods:**

This is a mixed methods, 2-phase interventional feasibility study at 2 Indian sites (Kalyani, West Bengal, and Chandigarh and Panchkula, North India), chosen to represent contrasting urban-planned and mixed urban-rural catchments. Phase 1 trains 40 community health and education workers (accredited social health activists, auxiliary nurse midwives, Anganwadi workers, and female teachers), who then deliver structured education to 912 adolescent girls in phase 2. A subset of 406 girls will be allocated to MCs or MPs and followed for 12 months via diaries, home visits, and focus group discussions. Quantitative data will be analyzed in SPSS; qualitative data will undergo reflexive thematic analysis in NVivo, guided by the Behavior Change Wheel and Capability, Opportunity, Motivation–Behavior framework. A cost-utility analysis using EQ-5D-Y and quality-adjusted life-years will assess cost-effectiveness from a societal perspective.

**Results:**

The study is funded by the Indian Council of Medical Research (proposal IIRPSG-2024-01-03552), with funding confirmed in March 2024. Ethics approval was obtained in January 2024 (Postgraduate Institute of Medical Education and Research, Chandigarh) and in April 2024 (All India Institute of Medical Sciences, Kalyani). Phase 1 sensitization was completed at both sites by September 2024, with all 40 workers trained (retention 100%). As of March 2026, 512 of 912 (56%) adolescent girls have completed phase 2 sensitization, and 210 of 406 (52%) have been enrolled and allocated to MCs or MPs in phase 3. Data collection is expected to conclude by December 2026, with analysis in early 2027 and outcome findings to be submitted for publication by late 2027.

**Conclusions:**

This feasibility study will generate evidence on the acceptability, usability, and cost-effectiveness of a community worker– and teacher-led model for promoting sustainable menstrual products among adolescent girls, informing scale-up strategies in similar low- and middle-income settings.

## Introduction

### Background

Menstrual hygiene management remains a critical health and equity concern for adolescent girls in India and other low- and middle-income countries. Although national schemes such as the Menstrual Hygiene Scheme and the Rashtriya Kishor Swasthya Karyakram have improved access to disposable sanitary pads, sustainable alternatives such as menstrual cups (MCs) and menstrual panties (MPs) have yet to gain significant traction [1,2]. According to National Family Health Survey–5 data, only 64% of adolescent girls aged 15‐19 years in India use hygienic menstrual protection, and reusable options are rarely adopted [2]. Of the approximately 1.42 billion people living in India today, 22.2% of women are in their reproductive years; these women use sanitary pads, tampons, MCs, and cloths during menstruation, and on average a woman generates around 150 kilograms of nonbiodegradable menstrual waste over her reproductive lifespan, with conventional sanitary pads taking 500‐800 years to decompose [3-6].

MCs and MPs are regarded as more economical and eco-friendly alternatives to conventional products [1]. MCs have a lifespan of up to 10 years, while MPs provide reusable absorbent underwear that lowers monthly expenses and avoids internal insertion, making both options particularly suitable for low-resource settings [3,7]. Despite these benefits, uptake of MCs and MPs in India remains below 5% in most published reports, reflecting deeply rooted menstrual taboos, ignorance, virginity-related fears, and misconceptions regarding usability and safety [1,8-10]. More recent single-site studies continue to document this gap: cross-sectional surveys among young women in South India in 2024‐2025 report that although a majority have heard of MCs, actual awareness of correct use and uptake remain poor, with unfavorable attitudes documented in over half of respondents [6,11].

Evidence from other settings indicates that adolescent girls are more receptive to sustainable products when interventions combine peer support, structured education, and trusted facilitator involvement [1,11,12]. When MCs and MPs are introduced with supervision and peer support, they are more often perceived as discreet, comfortable, and acceptable [1,13-16]. Nonetheless, a critical gap persists in large-scale, community-based interventions that jointly address awareness generation and product distribution, particularly in the Indian context. Community health workers—accredited social health activists (ASHAs), auxiliary nurse midwives (ANMs), and Anganwadi workers (AWWs)—together with female schoolteachers, are well positioned to promote behavioral change among adolescent girls because of their existing community trust and reach. Their involvement is critical for shifting narratives, offering accurate guidance, and normalizing the use of reusable menstrual products [9]. In this manuscript, we use the term “community health and education workers” (rather than the broader term “stakeholders”) to refer specifically to this frontline group of ASHAs, ANMs, AWWs, and female schoolteachers of grades 8‐12, who are the direct recipients of phase 1 training and the subsequent deliverers of phase 2 community education; the term “stakeholders” is reserved for this same group throughout the manuscript for consistency and is not intended to include policymakers or other actors not directly involved in intervention delivery.

A recent qualitative analysis of Indian adolescent girls’ perspectives on menstrual health promotion found that girls consistently identified trusted adult facilitators, peer discussion, and early, repeated exposure to accurate information as key enablers of healthier menstrual practices, reinforcing the rationale for a facilitator-led model such as the one proposed here [17]. Similarly, a narrative review of menstrual health interventions for adolescent girls in rural India highlighted that decentralized, community-driven models—particularly those embedded within existing government frontline worker cadres—tend to be more sustainable than vertically delivered, one-off campaigns [9].

As this intervention seeks to change health behavior at both the facilitator and adolescent levels, its design is informed by the Behavior Change Wheel and its central Capability, Opportunity, Motivation–Behavior (COM-B) model, which conceptualizes behavior as a function of capability, opportunity, and motivation [18]. Capability is targeted through structured knowledge- and skills-based training (menstrual physiology, product use, and troubleshooting); opportunity is addressed by ensuring physical access to free products, demonstration materials, and socially supportive group settings (eg, single-sex workshops and guardian engagement); and motivation is targeted through myth-busting, testimonial-based messaging, and peer modeling delivered by trusted community figures. This framework guided both the content of the phase 1 training curriculum and the phase 2 adolescent sensitization modules and will subsequently guide the interpretation of qualitative findings on barriers and facilitators to adoption.

The current study addresses this implementation and knowledge gap by developing and evaluating a community-based, facilitator-led intervention to promote MCs and MPs among adolescent girls, through the sensitization and training of community health and education workers. Through workshops, campaigns, and peer engagement, these facilitators will disseminate knowledge and foster positive attitudes toward sustainable products. The novelty of the study lies in the simultaneous evaluation of both products, integrating educational outreach with a cost-utility framework within a single feasibility study. Findings are expected to inform culturally sensitive scale-up strategies across similar low- and middle-income countries contexts. This protocol outlines a 2-phase interventional feasibility study to investigate the feasibility, acceptability, and scalability of this community-driven model in 2 Indian settings.

### Objectives

This study aims to evaluate the feasibility, acceptability, and effectiveness of a community-based model for promoting sustainable menstrual products—MCs and MPs—among adolescent girls in 2 urban and mixed urban-rural Indian settings.

The specific objectives are as follows:

1. To assess the impact of sensitization and training of community health and education workers on their knowledge, attitudes, and practices regarding sustainable menstrual hygiene products
2. To evaluate the effectiveness of facilitator-led community campaigns in enhancing adolescent girls’ awareness, knowledge, and readiness to adopt MCs and MPs
3. To examine the acceptability, challenges, and user experiences of adolescent girls using MCs and MPs over a defined 12-month period
4. To perform an economic evaluation of MCs and MPs in terms of cost-effectiveness, sustainability, and affordability

## Methods

### Study Design

This study is designed as a 2-phase, interventional feasibility study using a mixed methods, convergent-parallel approach, in which quantitative and qualitative data are collected concurrently in each phase and integrated at the interpretation stage [19]. Phase 1 focuses on sensitization and preintervention assessment of community health and education workers, while phase 2 evaluates the effectiveness and acceptability of MCs and MPs among adolescent girls through direct intervention and structured follow-up. As a feasibility study, the protocol has been developed with reference to the CONSORT (Consolidated Standards of Reporting Trials) 2010 extension for pilot and feasibility trials and relevant SPIRIT (Standard Protocol Items: Recommendations for Interventional Trials) guidance for reporting trial protocols, adapted for a mixed methods, nonrandomized community intervention design [20,21].

### Study Setting and Rationale for Site Selection

**Figure 1.**
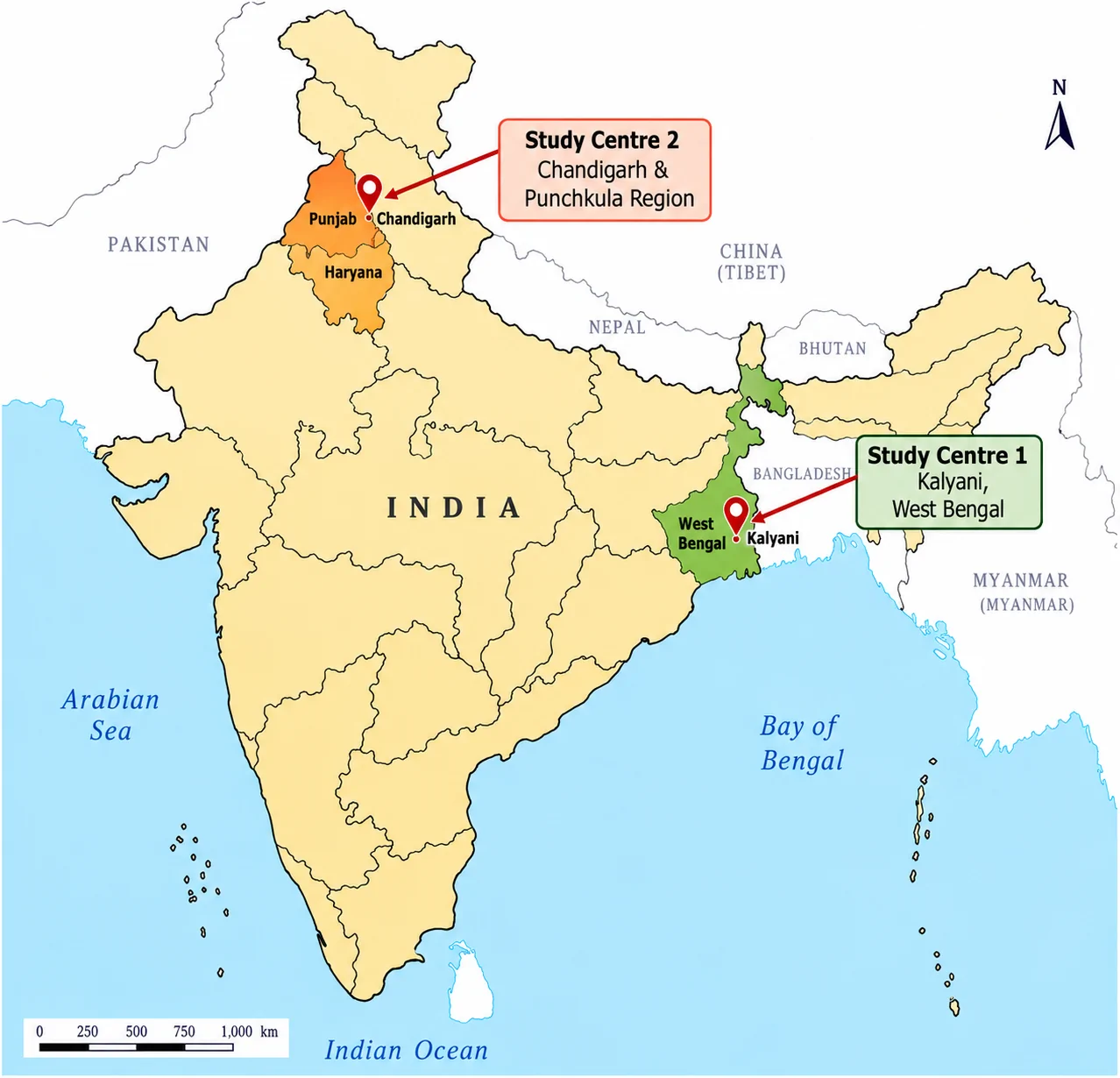
Map of India showing the 2 study centers.

The study will take place in 2 Indian sites, chosen to capture contrasting geographic and sociocultural contexts while retaining the feasibility of implementation within the study team’s existing institutional and field infrastructure (Figure 1):

- Center 1—East India (Kalyani, West Bengal): Kalyani is a planned urban township in the Nadia district of West Bengal, approximately 50 kilometers (31 miles) from Kolkata. It functions as both a municipality and a community development block, and includes surrounding semiurban and rural catchment areas served by the affiliated primary health centers, allowing the capture of both urban and semirural adolescent populations within a single site.
- Center 2—North India (Chandigarh and Panchkula): Chandigarh is a union territory serving as the joint capital of Haryana and Punjab, recognized for its planned urban infrastructure. The study will include defined urban service areas of Chandigarh (Indira Colony and Sectors 25, 49, 50, and 52), together with Panchkula district, Haryana, which includes peri-urban and rural blocks, to allow comparison of urban and rural uptake within the same site.

The 2 sites were selected, in preference to a larger number of sites, to allow intensive, high-fidelity delivery of the facilitator-training and adolescent-sensitization components within the resources available for a feasibility study, while still enabling comparison across an eastern, planned-urban context (Kalyani) and a northern, mixed urban-rural context (Chandigarh-Panchkula). Recruitment at each site is organized at the level of predefined clusters (schools and their linked Anganwadi and health subcenter catchments), reflecting the fact that stakeholders and adolescent girls are naturally grouped within schools and community health worker catchment areas rather than sampled as unrelated individuals; this clustering was accounted for in the sample size calculation (see below) via a design effect. This represents a refinement of the original grant proposal, in which site selection was described in less detail; following external review of the funded proposal, the protocol was revised to make the cluster structure explicit and to ensure that both urban and rural and peri-urban catchments are represented at each site, rather than urban areas only, in order to improve generalizability of findings across the rural-urban gradient.

### Study Participants

The study involves two key participant groups: (1) adolescent girls aged 13‐19 years (N=912) who were enrolled in schools or attended community centers and (2) community health and education workers (n=40; ASHAs, ANMs, AWWs, and female schoolteachers of grades 8‐12; Textbox 1).

Textbox 1. **Eligibility criteria.**

**Inclusion criteria**

- Community health and education workers
  - All auxiliary nurse midwives (ANMs), accredited social health activists (ASHAs), and Anganwadi workers (AWWs) working in the selected health centers
  - All female schoolteachers involved in teaching grades 8 to 12

- Adolescents
  - Adolescent girls within the age group of 13‐19 years
  - Adolescent girls who have attained menarche
  - Resident of the city for the last 6 months
  - Able to read and understand the local language or Hindi

**Exclusion criteria**

- Community health and education workers
  - Male schoolteachers
  - ANMs, ASHAs, AWWs, and teachers not providing consent
- Adolescents
  - Girls with disabilities or mental illness that would preclude participation in group sessions or self-administered questionnaires
  - Pregnant or lactating
  - Not providing assent or informed consent

### Study Flow and Sample Size Calculation

**Figure 2.**
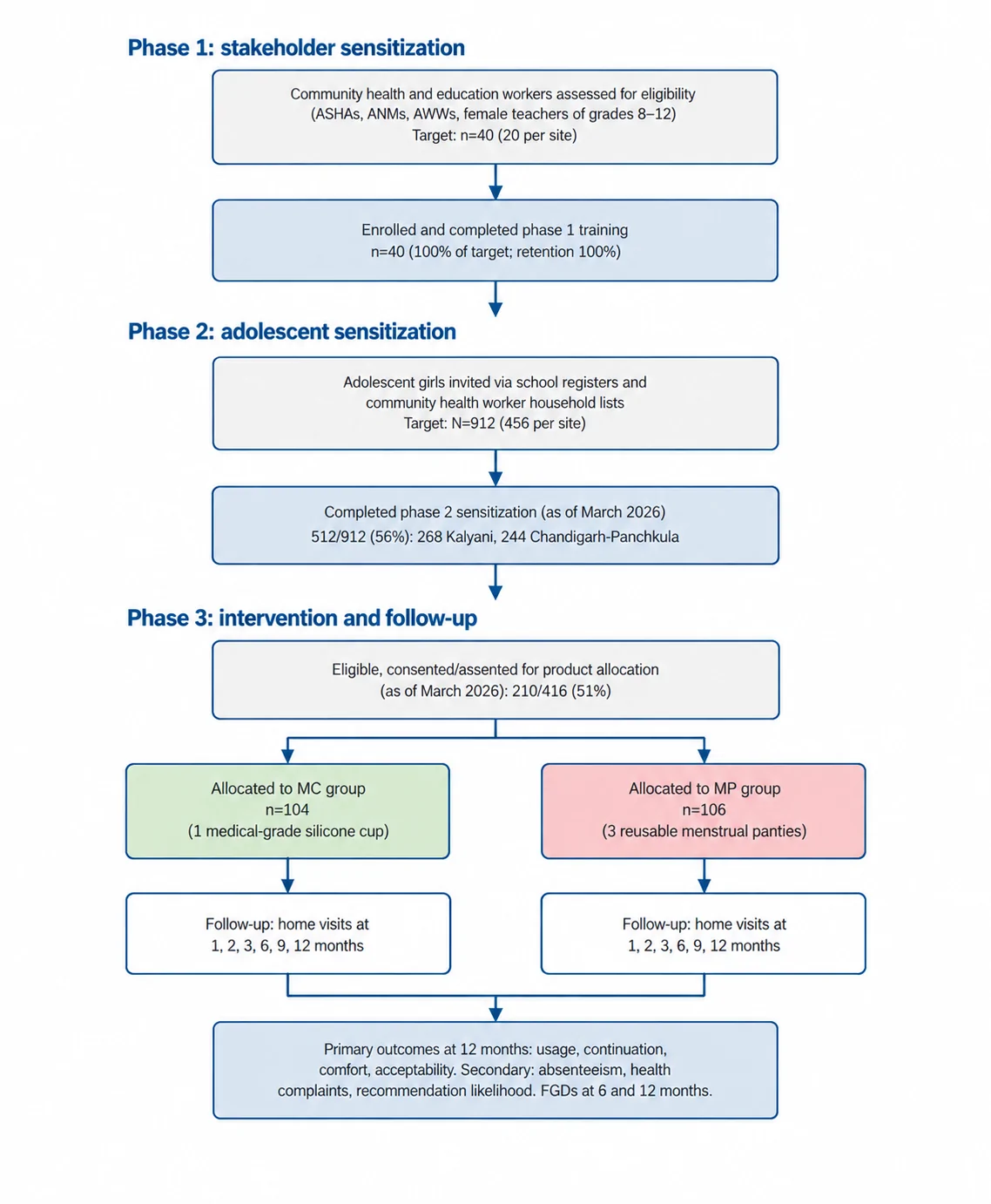
Participant flow across phase 1 (stakeholder sensitization), phase 2 (adolescent sensitization), and phase 3 (product allocation and 12-month follow-up), with enrollment and retention figures updated as of March 2026. ANM: auxiliary nurse midwife; ASHA: accredited social health activist; AWW: Anganwadi worker; FGD: focus group discussion; MC: menstrual cup.

Figure 2 depicts the overall participant flow across all 3 phases of the study, from eligibility assessment and enrollment of community health and education workers in phase 1, through adolescent sensitization in phase 2, to product allocation and 12-month follow-up in phase 3. The figure has been drawn as a single, simplified linear flow to reflect the status as of March 2026 (see Results). Boxes shaded gray denote eligibility and target populations at each phase; boxes shaded blue denote participants actually enrolled and retained; and the green and orange boxes in phase 3 denote the MC and MP allocation arms, respectively.

For the adolescent cohort, assuming a baseline sustainable-product usage rate of 5% and a projected postintervention uptake of 20%, with 80% power and 5% significance, the minimum required sample size was calculated as 304 adolescent girls per site using a standard formula for comparing 2-proportions formula. To account for clustering by school and community catchment, a design effect of 1.5 (based on an assumed intracluster correlation coefficient of 0.02 and an average cluster size of 25 girls, consistent with design effects reported in comparable school-based menstrual health cluster studies) was applied, giving a minimum requirement of 456 adolescent girls per site (total N=912) for the phase 2 sensitization cohort. Of these, a subgroup of 416 girls (n=208 per site) will be enrolled in the phase 3 product-use follow-up; this subgroup size was determined pragmatically in line with recommendations for feasibility and pilot studies, which emphasize that a fully powered comparative sample is not required at this stage but that sample size should still be justified in relation to the primary feasibility objectives (recruitment, retention, and product continuation) rather than a hypothesis-testing end point [22,23]. A minimum of 30‐35 participants per arm per site is considered adequate to estimate continuation and acceptability proportions with reasonable precision (95% CI width ≤±16%) for feasibility purposes, and the planned 203 girls per site substantially exceeds this threshold, allowing additional precision for the secondary comparison between MC and MP users. Twenty community health and education workers per site will be enrolled (total n=40) for training and postintervention assessment; this number reflects the total eligible cadre of ASHAs, ANMs, AWWs, and grade 8‐12 female teachers attached to the selected clusters at each site, and was not derived from a formal power calculation, consistent with standard practice for facilitator and trainer cohorts in implementation feasibility studies.

### Theoretical Framework

Intervention content and delivery are underpinned by the COM-B model and Behavior Change Wheel [18], which were selected because they explicitly link behavioral diagnosis (why uptake of MC and MP is currently low) to specific intervention functions (education, persuasion, training, modeling, and enablement) used in this study. Table 1 maps each COM-B component (Capability, Opportunity, and Motivation) to the corresponding intervention activities and outcome measures used in phases 1‐3.

**Table 1.** Mapping of Capability, Opportunity, Motivation–Behavior components to intervention activities and outcome measures.

COM-B^a^ component	Definition	Intervention activities (study phase)	Outcome measures
Capability–psychological	Knowledge and understanding needed to use MCs^b^ and MPs^c^ correctly	Structured training on menstrual physiology, product mechanics, and insertion or removal technique (phase 1 workshops; phase 2 adolescent sessions)	Pre-post knowledge scores (phase 1 and phase 2 questionnaires)
Capability–physical	Practical skill to insert, remove, and maintain the product	Hands-on demonstration using anatomical models; illustrated instructional booklets (phase 1 and phase 2)	Self-reported ease of insertion and removal (acceptability scale, phase 3)
Opportunity–physical	Access to free products, private facilities, and water for cleaning	Free product provision; instructional materials; tracking diaries (phase 3)	Product usage and continuation rates (phase 3 diaries and home visits)
Opportunity–social	Supportive family, peer, and school environment	Single-sex group workshops; guardian information sessions and FGDs^d^ (phase 2 and phase 3)	FGD themes on family or peer support (phase 2 and phase 3 FGDs)
Motivation–reflective	Conscious beliefs, attitudes, and perceived benefits or risks	Myth-busting content and testimonial-based messaging (phase 1 and phase 2 modules)	Attitude subscale scores (phase 1 and phase 2 questionnaires)
Motivation–automatic	Habitual and emotional responses, comfort, and stigma	Peer modeling delivered by trusted community figures (phase 2 sessions; phase 3 follow-up)	Acceptability scale; FGD themes on stigma or comfort (phase 3)

a COM-B: Capability, Opportunity, Motivation–Behavior.

b MC: menstrual cup.

c MP: menstrual panty.

d FGD: focus group discussion.

### Intervention Design and Development

#### Overview

Educational materials for both phases were developed by the study team (comprising public health physicians, a behavioral scientist, and community medicine faculty) through a 3-step process: (1) a rapid review of existing MC and MP education materials and relevant literature to identify core content domains (menstrual physiology, product mechanics, insertion or removal technique, hygiene and storage, and myth-busting); (2) drafting of a stakeholder training module (for phase 1) and a parallel, simplified adolescent-facing module (for phase 2), including presentation slides, an illustrated instructional booklet, insertion or removal demonstration videos using anatomical models, and posters for classroom and community display; and (3) content and face validation by an expert panel of 5 reviewers (2 gynecologists, 1 adolescent health specialist, 1 health educator, and 1 community health worker supervisor), followed by pilot testing of both modules with a convenience sample of 5 community health and education workers and ten adolescent girls (not included in the main study) at a site not used for the main study. Feedback from pilot testing was used to simplify language, add locally relevant illustrations, and adjust session length before finalizing both modules. All materials are available in English, Hindi, and Bengali.

#### Phase 1: Stakeholder Sensitization

##### Workshops

Workshops will be conducted with 40 community health and education workers to build knowledge of menstrual health, sustainable menstrual hygiene practices, MC and MP use, benefits, myths, and insertion and removal demonstrations, using the module described above. A toolkit including information, education, and communication materials, demonstration videos, and posters will be used across 2 half-day workshops per site; the workshops will be delivered by the study team. Pre-post assessments will evaluate changes in knowledge, attitude, and comfort with discussing the topic (see below). Trained community health and education workers will subsequently engage adolescent girls through interactive sessions in schools and community settings using the adolescent-facing module.

##### Presensitization and Postsensitization Assessments

A self-administered, structured questionnaire will be used to elicit information on sociodemographic variables; knowledge of puberty and menstruation; knowledge of menstrual health management; knowledge of sustainable menstrual products (MC, MP), their benefits, and correct use; and attitudes toward the adoption of MCs and panties. The questionnaire is not identical to the instruments used in the cited prior studies [6,24]; rather, it was developed by the study team using selected knowledge-domain and attitude-domain items adapted from those published instruments as a starting point, with the following changes: (1) items specific to the earlier studies’ college and medical-student populations were removed or reworded for an adolescent (grades 8‐12) reading level; (2) items on MPs (not covered in [6,24,25], which addressed MCs only) were newly developed by the study team; and (3) sociodemographic items relevant to this study’s rural and urban Indian sites (eg, school type and residence type) were added. The adapted instrument was pilot-tested for clarity and comprehension among 10 community health and education workers prior to use (Cronbach α for the knowledge subscale was 0.78 in pilot testing). The full study questionnaire (phase 1 stakeholder version and phase 2 adolescent version), with items mapped to their source (adapted vs newly developed), is provided as Multimedia Appendix 1. Feedback will also be collected on the resource materials developed for adolescent girls, to permit iterative refinement before phase 2 roll-out.

### Phase 2: Adolescent Sensitization and Recruitment

The sensitization intervention will be conducted in small-group workshops (approximately 20‐25 girls per group) using materials developed and validated in phase 1, covering menstrual health, hygiene, and correct usage of MCs and MPs. Sessions will be led by previously trained community health and education workers (ASHA, ANM, AWW, and female teachers), ensuring cultural sensitivity and local relevance, and will run for approximately 90 minutes across 2 sessions per group. Adolescents will be recruited as per the inclusion criteria above through school registers (for school-going girls) and community health worker household lists (for out-of-school girls), with information sessions held for parents and guardians prior to enrollment. Preintervention and postintervention questionnaires (structured, self-administered, in the local language) will measure changes in knowledge, attitudes, and perceived readiness to adopt MC and MP, using the same core knowledge and attitude domains as the phase 1 instrument, adapted to the adolescent literacy level. Additionally, 2 focus group discussions (FGDs) will be held at each site—one with adolescent girls and one with their female guardians—to explore barriers and facilitators to product adoption, using a semistructured topic guide based on the COM-B framework (Multimedia Appendix 2). Quantitative data will be analyzed using SPSS (version 26; IBM Corp), and qualitative data will undergo thematic analysis to develop a comprehensive understanding of the social and behavioral influences on sustainable menstrual product adoption.

### Phase 3: Intervention and Follow-up

A total of 416 adolescent girls aged 13‐19 years, equally divided between the 2 study sites, who complete the phase 2 sensitization and provide consent or assent for product use, will be allocated to either of the following:

- MC group: receives a size-appropriate medical-grade silicone MC with instructional manual.
- MP group: receives 3 reusable MPs with instructional pamphlets.

Allocation to the MC or MP group will be based on participant preference following a structured counselling session in which both products, their insertion or use requirements, and maintenance needs are explained; girls who express no strong preference will be allocated using a randomization sequence generated in blocks of 4, stratified by site, to achieve approximately equal group sizes. This preference-informed allocation approach was chosen, rather than full randomization, because acceptability and correct use of MCs in particular are strongly influenced by willingness to use an internally inserted product, and forcing product allocation against strong preference was judged likely to compromise both retention and the validity of acceptability outcomes in a feasibility study of this nature; this deviation from a purely randomized design, and its rationale, is reported here transparently as recommended for feasibility study reporting [20].

Participants will receive free products along with instructional materials and menstrual-tracking diaries that record menstruation dates, school attendance, side effects, and usage challenges. Monthly follow-ups will occur through home visits at 1, 2, 3, 6, 9, and 12 months, conducted by trained community health and education workers using a structured follow-up checklist.

A 12-month follow-up duration was chosen in line with prior longitudinal descriptive work showing that adaptability and correct use of MCs typically stabilize only after several menstrual cycles of sustained use and support [25].

Primary outcomes are product usage, continuation (proportion still using the allocated product at 12 mo), comfort, and acceptability (assessed using a structured acceptability scale administered at 1, 3, 6, and 12 mo, capturing ease of insertion or removal, comfort during daily activities, leakage experience, and overall satisfaction). Secondary outcomes include school absenteeism during menstruation, menstruation-related health complaints, and likelihood of recommending the product to others. FGDs conducted at 6 and 12 months will further explore facilitators and barriers to continued use.

### Rigor and Trustworthiness of Qualitative Methods

Qualitative data collection and analysis will follow a reflexive thematic analysis approach as described by Braun and Clarke [26], which emphasizes the researcher’s active, situated role in generating themes rather than treating themes as simply “emerging” from the data. All FGDs will be conducted by 2 trained qualitative researchers (a moderator and a note-taker), audio-recorded with consent, transcribed verbatim, and translated into English where necessary, with back-translation of a 10% sample to check accuracy. Coding will be conducted independently by 2 researchers using NVivo (v14, Lumivero), with a third senior researcher reviewing coding decisions and resolving discrepancies through discussion; reflexive memos will be maintained throughout to document analytic decisions and the researchers’ own positionality (as public health professionals external to the immediate community) and its potential influence on interpretation. Trustworthiness will be addressed using the criteria of credibility (through investigator triangulation, member-checking of preliminary themes with a subset of participants, and prolonged engagement through the 12-month follow-up), dependability (through an audit trail of coding decisions and analytic memos), confirmability (through independent coding and reflexive discussion to limit individual researcher bias), and transferability (through thick description of the study contexts to allow readers to judge applicability to other settings) [27].

### Mixed Methods Integration

Quantitative and qualitative data will be integrated at the design, methods, and interpretation levels using a convergent-parallel mixed methods approach [19]. At the design level, qualitative FGD findings and quantitative questionnaire data are collected concurrently within each phase rather than sequentially. At the methods level, integration will occur through “merging”—quantitative results (eg, proportions reporting continued use and comfort scores) and qualitative themes (eg, reasons for discontinuation and social barriers) will be displayed side-by-side in joint display tables organized by outcome domain (uptake, continuation, comfort or acceptability, and barriers) to identify areas of convergence, divergence, or expansion between the 2 data sources. At the interpretation level, a triangulation protocol will be used in which discrepancies between quantitative and qualitative findings (eg, high self-reported continuation alongside qualitative reports of discomfort) will be explicitly examined and reported rather than resolved by preferring one data source over the other. This directly addresses triangulation as a stated strength of the study design (see Discussion) by specifying, in advance, how it will be operationalized.

### Representativeness and Subgroup Considerations

Given that grant-stage external review previously highlighted the importance of rural-urban variation, recruitment records will capture each participant’s residence type (urban, peri-urban, or rural, based on the Census of India classification) and school type (government, government-aided, or private, where applicable) at both sites. Sample composition by residence type and school type will be reported descriptively and used, where numbers permit, as covariates in the multivariate regression models described below, to allow exploration of whether uptake and continuation differ between urban and rural or peri-urban participants. Given the feasibility-study sample size, these subgroup analyses will be considered hypothesis-generating rather than confirmatory.

### Data Analysis

Quantitative data will be analyzed using SPSS (v25). Descriptive statistics (frequencies, means, and SDs) will be used to summarize participant characteristics and uptake. Paired 2-tailed *t* tests and chi-square tests will evaluate pre- and postintervention differences in knowledge, attitude, and practice scores. Multivariate logistic regression will examine predictors of product adoption and continuation, including site, residence type (urban or rural), age, product type (MC vs MP), and baseline knowledge and attitude scores, with statistical significance set at *P*<.05. Qualitative data will be analyzed thematically using NVivo (v14), following the 6-phase reflexive thematic analysis approach described above.

### Economic Evaluation

A cost-utility analysis will be undertaken from a societal perspective. Direct costs (product cost, training, distribution, and support) and indirect costs (time lost by participants and facilitators, perceived burden) will be collected using structured costing proformas completed by the study team and, for indirect costs, by participants via a tracking diary. Health-related quality of life will be assessed using the EQ-5D-Y, a generic, 5-dimensional health status measure developed specifically for use in children and adolescents [28]. The EQ-5D-Y has demonstrated acceptable feasibility, test-retest reliability (agreement 70%‐99% across dimensions), and known-groups validity in multinational pediatric and adolescent samples [29]. Reliability and content-validity data specific to Indian adolescents remain limited; a recent Indian study piloting the EQ-5D-Y-3L among school-going adolescents reported generally good feasibility but noted comprehension difficulty with the “having pain or discomfort” item and the “today” time-frame among some participants [30]. In light of this, the study team will conduct cognitive debriefing of the Hindi or Bengali translations of the EQ-5D-Y with a small sample of adolescent girls (n=10 per site) before baseline administration, and any comprehension difficulties encountered will be reported alongside the health-related quality of life findings. The EQ-5D-Y will be administered at baseline, 6 months, and 12 months. The incremental cost-effectiveness ratio will be calculated for MCs and MPs relative to usual practice (continued use of disposable pads) in terms of cost per quality-adjusted life-year gained, and results will be presented separately for each site to reflect potential differences in local costs.

### Ethical Considerations

#### Ethics Approval

The study protocol has been reviewed and approved by the Institutional Ethics Committee (IEC) of the Postgraduate Institute of Medical Education and Research, Chandigarh (application and approval reference number: IEC-I012024-3219; approved January 2024), and by the Institutional Ethics Committee of the All India Institute of Medical Sciences (AIIMS), Kalyani (application and approval reference or certificate number: IEC/AIIMS/Kalyani/certificate/2024/321; approved April 2024). Both reference numbers serve as the unique application and approval identifiers issued by the respective committees; no separate protocol registration number has been issued.

#### Participants’ Consent

Written informed consent will be obtained from all participants aged 18 years and above. For those under 18 years, written assent will be sought, in addition to consent from a parent or legal guardian, prior to participation. Participants will be informed that participation is voluntary and that they may withdraw at any stage without consequence. All data will be handled in accordance with strict confidentiality and data protection protocols. All identifiable information will be anonymized, and only deidentified data will be used for analysis and publication.

### Dissemination Plans

Findings will be disseminated via peer-reviewed journals, national and international conference presentations, and policy briefs for distribution to public health authorities and relevant stakeholders. Community-level dissemination sessions will also be organized to return results to participants and local decision-makers. Collaborations with educational institutions, health care providers, and policymakers will be sought to incorporate the project’s findings into existing menstrual health initiatives. A plan will also be developed to address any identified challenges in the adoption of sustainable menstrual products, aiming for broader acceptance and use.

## Results

This protocol reports on a study that is funded and currently being implemented. The study was funded by the Indian Council of Medical Research in March 2024 (Proposal ID: IIRPSG-2024-01-03552). Ethics approval was granted by the Postgraduate Institute of Medical Education and Research Chandigarh Institutional Ethics Committee in January 2024 and by the AIIMS Kalyani Institutional Ethics Committee in April 2024.

Phase 1 stakeholder sensitization workshops were completed at both sites between July and September 2024. All 40 targeted community health and education workers (20 per site) were enrolled and completed training, giving a phase 1 retention rate of 100%. Pre-post assessment of this cohort showed an increase in mean knowledge scores following training; a full analysis of these phase 1 outcomes will be reported separately once phase 2 data collection is complete, consistent with journal guidance to avoid reporting substantive analytic results at the protocol stage.

Phase 2 adolescent sensitization commenced in October 2024. As of March 2026, 512 of the planned 912 (56%; n=268 in Kalyani and n=244 in Chandigarh-Panchkula) adolescent girls have completed baseline sensitization sessions. Of these, 210 of the planned 416 (51%) girls eligible for phase 3 have provided consent or assent and have been allocated to the MC (n=104) or MP (n=106) group; enrollment in the remaining clusters is scheduled to continue through 2026.

One deviation from the original grant proposal has occurred to date: the Chandigarh site catchment was expanded to include the district of Panchkula, Haryana, in addition to the originally proposed urban Chandigarh sectors, in order to incorporate a peri-urban or rural comparison population within the same site, as detailed in the Study Setting section above. This change was approved by both ethics committees as a minor protocol amendment prior to the start of phase 2 recruitment at that site.

Data collection for phase 3 follow-up is expected to continue through December 2026. Data analysis is planned for the first quarter of 2027, with submission of substantive outcome findings for peer-reviewed publication anticipated by late 2027.

## Discussion

### Implications and Expected Findings

Despite growing awareness of the health, financial, and environmental benefits of reusable menstrual products such as MCs and MPs, their adoption in India has remained relatively low [1,9]. Our 2-phase, community-based model specifically empowers trusted frontline workers (ASHAs, ANMs, AWWs, and teachers) to provide adolescents with peer support and consistent education, thereby directly addressing barriers, such as cultural taboos, misinformation, and a lack of safe-use guidance, in line with the Capability-Opportunity-Motivation pathways specified in our theoretical framework [18].

There is evidence that peer-led and structured educational interventions substantially increase MC uptake. In a pilot study undertaken in Kerala, 50% of participants reported willingness to use an MC after receiving structured awareness education, and a study conducted in Nepal found that adolescents who received structured counseling were 60% more likely to accept the product [1,13,14]. Furthermore, despite being noninvasive and appealing to teenagers, reusable products such as MPs remain underused; for example, in a study conducted in Australia, only 24% of respondents reported using period underwear during their last menstrual period, and despite generally positive experiences, challenges such as limited information, upfront cost, and cleaning requirements were reported to hinder wider adoption [11].

The mixed methods design of this study allows triangulation of quantitative outcomes (eg, adoption rates, differences between MC and MP users) with qualitative insights into usability, stigma reduction, and cultural acceptability, using the prespecified joint-display and triangulation-protocol approach described above [19]. The inclusion of cost-utility analysis will provide data on affordability and scalability, aspects often missing from previous interventions [31]. If found to be feasible and acceptable at both sites, the intervention model has potential for scale-up through existing public health and school systems, contributing to menstrual equity, environmental sustainability, and the empowerment of adolescent girls.

### Strengths and Limitations

To our knowledge, this is the first study in India to evaluate both MCs and MPs through a community-driven, adolescent-focused intervention. It adopts a robust mixed methods design that integrates facilitator involvement, an explicit behavior-change theoretical framework, and a cost-effectiveness evaluation, with a prespecified approach to qualitative rigor and mixed methods integration. The engagement of grassroots health workers and educators enhances the contextual relevance and community reach of the study, and the inclusion of both urban and rural or peri-urban catchments at each site strengthens generalizability relative to earlier single-context studies. However, potential limitations include cultural resistance to product use, the risk of participant attrition over the 12-month follow-up period, the use of preference-informed rather than fully randomized product allocation (which may introduce selection differences between MC and MP groups), and limited generalizability beyond the 2 geographic sites studied.

## Supplementary material

10.2196/93628 Multimedia Appendix 1 Study questionnaire.

10.2196/93628 Multimedia Appendix 2 Focus group discussion topic guide.

10.2196/93628 Peer Review Report 1 Peer review report by the Indian Council of Medical Research.

## Abbreviations

AIIMS: All India Institute of Medical Sciences
ANM: auxiliary nurse midwife
ASHA: accredited social health activist
AWW: Anganwadi worker
COM-B: Capability, Opportunity, Motivation–Behavior
CONSORT: Consolidated Standards of Reporting Trials
FGD: focus group discussion
IEC: Institutional Ethics Committee
MC: menstrual cup
MP: menstrual panty
SPIRIT: Standard Protocol Items: Recommendations for Interventional Trials
