# Supplementary material for: Developing a Community-Based Model for the Adoption of Sustainable Menstrual Products Among Adolescent Girls: Protocol for a 2-Phase Interventional Feasibility Study
Source: JMIR Res Protoc. 2026 Sep 22;15:e93628. doi: 10.2196/93628 (PMC13597021; doi:10.2196/93628)
Supplement: Supplementary file 1 [file resprot-v15-e93628-s003.pdf]

Gmail

Rekha Dutt <rekha.cmfm@aiimskalyani.edu.in>

ICMR: Proposal Id IIRPSG-2024-01-03552 result (no-reply to this email)

4 messages

no-reply@hq.icmr.org.in <no-reply@hq.icmr.org.in> Mon, Oct 14, 2024 at 12:20 PM
To: Dr Rekha Dutt <rekha.cmfm@aiimskalyani.edu.in>
Cc: ICMR-ePMS <po.epms@icmr.gov.in>, ICMR-ePMS <no-reply@hq.icmr.org.in>

Dear Dr Rekha Dutt,

This is with reference to your proposal Id “**IIRPSG-2024-01-03552**”, titled “**Developing a Community-Based Model for the Adoption of Sustainable Menstrual Products among Adolescent Girls: A Two-Phase Interventional Feasibility Study**”.

We are pleased to inform you that your project has been approved, subject to satisfactory response to the following technical and budgetary comments (as applicable).

Technical Comments:
• Detailed justification regarding sample size estimation is required. Since this will be cluster based study, cluster specific measures should be included in samples size like design effect or intracluster correlation coefficient, this is likely to increase the sample size.
• The outcome measures are not clear based on which sample size is computed, especially objective 2 in deciding number of schools and each school (cluster), number of students required.
• More number of schools should be included to gain more heterogeneity, as novel approaches will be utilized, so feasability/acceptability in different settings should be examined.
• Strata of urban and rural population should also be considered.
• Product Quality may be considered/checked for sustainable products through some lab testing etc.
• The cost-effectiveness of the intervention could be evaluated by reviewing existing research.
• Inter-disciplinary collaborators are missing, within two weeks of receipt of this email, please upload a revised proposal addressing the technical comments. For ease of review do highlight the revisions.

Budgetary Comments: NIL, PI to prepare a justification of budget and response to budgetary comments for a VC meeting with Budget Review Committee to be held within the next two weeks of receipt of the email.

We would also ask you to urgently arrange for all the CODAL formalities to be completed as per the current Guidelines for Extramural Research Programme of ICMR. ***Please submit for IEC/ regulatory approvals (as applicable) immediately.***

If the documents pertaining to CODAL formalities are not uploaded in ICMR-ePMS portal within the stipulated period (not later than 3 months of this email),the approval may be cancelled.

For any clarification/s on the technical or budgetary comments, please contact "Dr Saurabh Sharma (sharmas@icmr.gov.in)"

Important Note: This is an automated email. Kindly refrain from replying to this email.

Warm Regards
Program Officer

e-Project Management System
Indian Council of Medical Research
Department of Health Research
Ministry of Health & Family Welfare
Government of India
Ansari Nagar, New Delhi - 110029, India
www.icmr.gov.in

Gmail

Rekha Dutt <rekha.cmfm@aiimskalyani.edu.in>

Regarding Budget details to be submitted to ICMR

Saurabh Sharma <sharmas@icmr.gov.in> Wed, Dec 4, 2024 at 6:01 PM
To: Rekha Dutt <rekha.cmfm@aiimskalyani.edu.in>
Cc: Ashoo Grover <grovera.hq@icmr.gov.in>, ashoogrover <ashoogrover@gmail.com>, skhushwant910 <skhushwant910@gmail.com>, harleenkr92 <harleenkr.92@gmail.com>

Dear Dr. Rekha Dutt

With reference to your project proposal entitled "Developing a Community-Based Model for the Adoption of Sustainable Menstrual Products among Adolescent Girls: A Two-Phase Interventional Feasibility Study", IIRPSG-2024-01-03552 has received technical approval from the competent authority. As a next step, the proposal is scheduled to be reviewed by the Budget Review Committee. Therefore, you are requested to provide the necessary details in the template attached (PowerPoint) within one week.

Kindly contact me for any further queries.

Warm regards,
Dr. Saurabh Sharma,
MBBS, MD (Community Medicine),PGDMRCH
Scientist-D,
Delivery Research Division,
Indian Council of Medical Research,
Ansari Nagar, New Delhi, 110029
Mob No - +91 - 7840870009

Budget_PI_template_SG_2024.pptx
359K

Gmail

Rekha Dutt <rekha.cmfm@aiimskalyani.edu.in>

Sanction Letter & Grant Letter of first year of the ICMR project entitled: "Developing a Community-Based Model for the Adoption of Sustainable Menstrual Products among Adolescent Girls: A Two-Phase Interventional Feasibility Study"

Vipin Gupta <gvipin0545@gmail.com> Fri, Jan 24, 2025 at 4:47 PM
To: rekha.cmfm@aiimskalyani.edu.in
Cc: Saurabh Sharma <ssharmadoc@gmail.com>, vk.sapra@gmail.com, khushwant singh <skhushwant910@gmail.com>

Dear Ma'am,

Please find attached the sanction letter and grant letter of the first year of the ICMR Project: "IIRPSG-2024-01-03552 : Developing a Community-Based Model for the Adoption of Sustainable Menstrual Products among Adolescent Girls: A Two-Phase Interventional Feasibility Study".

The grant will be released within due course.

This is for your kind information and office record.

Please acknowledge the same.

Vipin Gupta
Project Technical Support-III
Division of Delivery Research
ICMR, HeadQuarter
New Delhi

Dr. Rekha Dutt.pdf
3840K
