# Supplementary material for: Developing a Community-Based Model for the Adoption of Sustainable Menstrual Products Among Adolescent Girls: Protocol for a 2-Phase Interventional Feasibility Study
Source: JMIR Res Protoc. 2026 Sep 22;15:e93628. doi: 10.2196/93628 (PMC13597021; doi:10.2196/93628)
Supplement: Supplementary file 2 [file resprot-v15-e93628-s001.docx]

## Study Questionnaire

**Accompanying manuscript:** Developing a Community-Based Model for the Adoption of Sustainable Menstrual Products among Adolescent Girls: Protocol for a Two-Phase Interventional Feasibility Study (JRP ms#93628)

This appendix provides the core content domains and representative items of the structured, self-administered questionnaire used in Phase 1 (community health and education workers) and Phase 2 (adolescent girls), administered pre- and post-sensitisation. Items are mapped to their source: **Adapted** items are drawn, with modification, from previously published menstrual cup knowledge-attitude-practice (KAP) instruments [Mogallapalli et al. 2025; Devi et al. 2025]; **New** items were developed by the study team for this protocol (in particular, all menstrual panty items, since the cited source instruments addressed menstrual cups only). The full instrument is administered in English, Hindi, or Bengali according to site and participant preference.

### Part A: Socio-Demographic Information (New)

- Age (years)
- Site (Kalyani / Chandigarh / Panchkula)
- Residence type (urban / peri-urban / rural, per Census of India classification)
- School type (government / government-aided / private / not currently in school), where applicable
- Grade/class (adolescent version only) or occupation (ASHA / ANM / AWW / teacher; stakeholder version only)
- Religion, caste category (optional, for descriptive purposes only)

### Part B: Knowledge of Puberty and Menstruation (Adapted)

- “Menstruation is a normal biological process.” (True/False/Don’t know)
- “A girl can attain menarche any time between 9 and 16 years of age.” (True/False/Don’t know)
- Open item: “In your own words, what causes menstruation to occur?”

### Part C: Knowledge of Menstrual Health Management and Sustainable Products (Adapted, with new MP-specific items)

- “A menstrual cup is a small, flexible cup made of medical-grade silicone that is inserted into the vagina to collect (not absorb) menstrual blood.” (True/False/Don’t know) — *Adapted*
- “A menstrual cup can be reused for several years if cleaned properly.” (True/False/Don’t know) — *Adapted*
- “Menstrual panties are reusable underwear with built-in absorbent layers that can replace disposable pads.” (True/False/Don’t know) — *New*
- “Menstrual panties need to be washed and dried before reuse, similar to regular cloth.” (True/False/Don’t know) — *New*
- “Using a menstrual cup can affect virginity or cause harm if used correctly.” (True/False/Don’t know; reverse-scored) — *Adapted*

### Part D: Attitude Towards Adoption of MC and MP (Adapted, extended to cover MP)

Items rated on a 5-point Likert scale (Strongly Disagree to Strongly Agree):

- “I would be comfortable trying a menstrual cup if guided by a trained health worker.” — *Adapted*
- “I would be comfortable trying menstrual panties as an alternative to disposable pads.” — *New*
- “My family would support my use of a menstrual cup/menstrual panty.” — *Adapted*
- “I am concerned about what my friends/community would think if I used a menstrual cup/menstrual panty.” — *Adapted*

### Part E: Practice / Readiness Items (Phase 2, adolescent version only) (New)

- “Have you ever seen or heard of a menstrual cup before this session?” (Yes/No)
- “Have you ever seen or heard of menstrual panties before this session?” (Yes/No)
- “How likely are you to try a menstrual cup or menstrual panty in the next 3 months?” (5-point scale, Very unlikely to Very likely)

### Part F: Phase 3 Acceptability Scale (administered at 1, 3, 6, and 12 months to product users only) (New)

Items rated on a 5-point scale (Very difficult/uncomfortable to Very easy/comfortable):

- Ease of insertion/removal (MC group only)
- Comfort during daily activities (school, sports, sleep)
- Experience of leakage in the past month (none / mild / moderate / severe)
- Overall satisfaction with the product
- Likelihood of continuing to use the product
- Likelihood of recommending the product to a friend or family member

### Psychometric Note

The knowledge subscale (Part C) showed a Cronbach’s alpha of 0.78 on pilot testing among ten community health/education workers. Full item-level psychometric analysis (including the attitude and acceptability subscales) will be conducted on Phase 1 and Phase 2 baseline data and reported alongside the primary outcome findings.
