# Supplementary material for: Developing a Community-Based Model for the Adoption of Sustainable Menstrual Products Among Adolescent Girls: Protocol for a 2-Phase Interventional Feasibility Study
Source: JMIR Res Protoc. 2026 Sep 22;15:e93628. doi: 10.2196/93628 (PMC13597021; doi:10.2196/93628)
Supplement: Supplementary file 3 [file resprot-v15-e93628-s002.docx]

## Focus Group Discussion Topic Guide

**Accompanying manuscript:** Developing a Community-Based Model for the Adoption of Sustainable Menstrual Products among Adolescent Girls: Protocol for a Two-Phase Interventional Feasibility Study (JRP ms#93628)

**Purpose:** To explore barriers, facilitators, and experiences relevant to the adoption of menstrual cups (MC) and menstrual panties (MP), organised around the Capability-Opportunity-Motivation (COM-B) framework. Separate, lightly adapted versions are used for (a) adolescent girls and (b) female guardians.

### A. Introduction (all groups)

1. Can you tell me a little about what you already knew about menstrual cups/panties before this study?

### B. Capability (knowledge and skills)

2. What did you find easy or difficult to understand about how to use an MC/MP?
3. What, if anything, made you feel confident (or not confident) about using the product correctly?

### C. Opportunity (physical and social environment)

4. What has made it easier or harder to use the product day-to-day (e.g., privacy, water access, school facilities)?
5. How have family members, friends, or teachers reacted to your use of the product? (Guardian version: How have you supported, or found it difficult to support, your daughter’s use of the product?)

### D. Motivation (beliefs, attitudes, social norms)

6. What concerns, if any, did you have before starting, and have these changed?
7. What would make you more or less likely to keep using this product / recommend it to others?

### E. Closing

8. Is there anything about your experience with the product, or with this programme, that we have not asked about but that you think is important for us to know?

Prompts and follow-up probes will be used flexibly by the moderator to explore emerging issues, consistent with a reflexive thematic analysis approach (Braun & Clarke, 2023).
